# A Low-Cost Continuous Turbidity Monitor

**DOI:** 10.3390/s19143039

**Published:** 2019-07-10

**Authors:** David Gillett, Alan Marchiori

**Affiliations:** 1 Department of Chemical Engineering, Bucknell University, Lewisburg, PA 17837, USA; 2 Department of Computer Science, Bucknell University, Lewisburg, PA 17837, USA

**Keywords:** turbidity, low-cost, continuous water quality monitor, water

## Abstract

Turbidity describes the cloudiness, or clarity, of a liquid. It is a principal indicator of water quality, sensitive to any suspended solids present. Prior work has identified the lack of low-cost turbidity monitoring as a significant hurdle to overcome to improve water quality in many domains, especially in the developing world. Low-cost hand-held benchtop meters have been proposed. This work adapts and verifies the technology for continuous monitoring. Lab tests show the low-cost continuous monitor can achieve 1 nephelometric turbidity unit (NTU) accuracy in the range 0–100 NTU and costs approximately 64 USD in components to construct. This level of accuracy yields useful and actionable data about water quality and may be sufficient in certain applications where cost is a primary constraint. A 38-day continuous monitoring trial, including a step change in turbidity, showed promising results with a median error of 0.45 and 1.40 NTU for two different monitors. However, some noise was present in the readings resulting in a standard deviation of 1.90 and 6.55 NTU, respectively. The cause was primarily attributed to ambient light and bubbles in the piping. By controlling these noise sources, we believe the low-cost continuous turbidity monitor could be a useful tool in multiple domains.

## 1. Introduction

The United Nations states that high-quality drinking water is at the core of sustainable development and is critical for socioeconomic development, healthy ecosystems, and for human survival itself. It is vital for reducing the global burden of disease and improving the health, welfare, and productivity of human populations. It is central to the production and preservation of a host of benefits and services for people. Water is also at the heart of adaptation to climate change, serving as the crucial link between the climate system, human society, and the environment [1].

Water quality monitoring is the process by which critical characteristics of water (physical, chemical, biological) are measured. Turbidity is one of the most universal metrics of water quality. It is a measure of the cloudiness (the inverse of clarity) of water. In watersheds, the presence of high turbidity can be indicative of both organic and inorganic materials. In the case of organic materials, high turbidity can indicate problems such as increased algae growth caused by fertilizer run-off. In the case of inorganic materials, high turbidity can indicate problems such as high suspended sediment caused by erosion during a rainstorm or water churn caused by high winds. Turbidity is a non-specific measure and therefore alone cannot identify the root cause of water cloudiness. However, under certain conditions, it can be used to estimate certain quantitative parameters such as stream loading, total suspended solids, and soil loss. There is a variety of published research on the effect of turbidity on different organisms and the implications on human drinking water [2,3,4].

Therefore, turbidity is a useful measure for many water resource management applications. This monitoring can help inform decisions regarding the allocation of funds and what future actions would be the best for a watershed. Presently, the sensors that are used are expensive, typically costing thousands of dollars. This causes most of the sensors to be owned by companies that communities hire to take samples a small number of times a year. This is far from the best approach as rapid changes in turbidity are indicative of problems. The best time to tackle these problems is right when they occur. With current sampling frequency, these rapid changes are unlikely to be measured and extremely unlikely to have proper actions taken to deal with the root cause of these changes. The key to efficient and proactive water resource management is continuous and accurate monitoring. However, the cost and complexity of deploying such monitoring systems presently limit their use. It is critical that the cost of individual sensors be decreased to make widespread implementations of these monitoring systems feasible. Also, it is critical that the accuracy of these sensors be high enough to provide useful water quality data. Automated continuous sensing would allow the labor cost of water monitoring to decrease substantially as after the initial setup, except for minor ongoing maintenance, the sensors run continuously without human intervention. An automated sensor platform could also be used by people with little, if any, formal training in water monitoring.

Open-source technologies have been identified as the most promising solution to this challenge [5]. As a result, some groups have begun developing their own low-cost monitoring solutions [6,7,8,9]. However, these prior works for turbidity monitoring focus on hand-held meters and leave continuous monitoring for future work. Lambrou et al. [10] built a complete continuous monitoring system using off-the-shelf sensors without addressing cost or complexity concerns. Lorena Parra et al. built a low-cost water quality monitoring system for fish farming that contained a simple turbidity sensor [11]. Kofi Sarpong Adu-Manu et al. [12] broadly review methods for water quality monitoring focusing on technologically advanced methods employing wireless sensor networks. In this paper, we present the development of a low-cost continuous turbidity sensor. Our goal is a sensor that could be used in both watershed and drinking water continuous monitoring applications.

## 2. Related Work

Standard laboratory methods to measure turbidity are well understood and the most commonly used standard is maintained as method 180.1 by the U.S. EPA [13]. This method specifies a tungsten lamp illuminating a sample from not more than 10 cm away with a photo-electric detector oriented 90${}^{\circ }$ from the source. This method is specified from 0–40 nephelometric turbidity units (NTU) with instrument sensitivity of at least 0.02 NTU in water under 1.0 NTU. The NTU units themselves are defined by the response of the nephelometric sensor to known standards. There is no mathematical definition of NTU.

There are at least four other standards for measuring turbidity using nephelometry (ISO 7027, GLI Method 2, Hatch Method 101033, and Standard Methods 2130B) [14]. These variants specify different light sources and detector arrangements. However, none of these standard methods lend themselves to low-cost continuous water quality monitoring. In this work, we follow the general approach of using a light source with a detector located at 90${}^{\circ }$ built using only commonly available electronic components, 3D printable structures, and open-source software with the goal of determining if such a low-cost sensor could be suitable for continuous water quality monitoring applications.

To our knowledge, Christopher Kelly and his team proposed the first low-cost turbidity sensor [8]. This project represents the first publicly available peer-reviewed characterization of an affordable nephelometric turbidimeter. The team set out to create a battery-powered, high accuracy turbidity meter for drinking water monitoring in low-resource communities. This goal required a few design constraints that they set out to meet: run on a single set of batteries for weeks to months of regular use, a high measurement accuracy, and the ability to differentiate small changes in turbidity especially over the range of 0–10 NTU, the sensor must have all of its parts documented and be able to be made by non-experts who want to create their own version of the sensor.

The developed system is a cuvette-based turbidity meter using a single near infrared light emitting diode and a TSL230R light-to-frequency sensor set at 90${}^{\circ }$ apart in a single beam design. This is where there are a single LED emitter and a single receiver perpendicular to the light beam from the LED. The receiver converts light intensity to a signal that can be read by a microcontroller. The theory behind this design is that the clearer the solution, the more light that makes it straight through the solution. The more turbid the solution, the more light that is reflected perpendicular to the light beam. The meter does not store the data but rather displays it on a LED display for manual recording. Using turbidity standards created using cutting oil and water, the team tested a known turbidity meter next to the created turbidity sensor and measured the readings from both. This data was used to create four calibration curves (each for a different range) that are used to convert the light-to-frequency sensor output from the created turbidity meter to the turbidity reported by the commercial sensor.

The study showed the created turbidity meter had an accuracy within 3% of the commercial sensor or 0.3 NTU whichever is larger over the range of 0.02 NTU to 1100 NTU. They reported that in 8 trials results were within 0.01 NTU for the four turbidity standards under 0.5 NTU. These results support the notion that a low-cost turbidity meter is a possibility; however, more tests to evaluate and verify these results are needed. The proposed next steps as of when the paper was written were to account for thermal fluctuation effects on the turbidity of a solution, minimizing the light leakage into the sensor housing through the external casing, investigating the use of GSM data transmission, and investigating an inline immersible version of the turbidity meter.

Closely related is the optical sensor for sea quality monitoring by Filippo Attivissimo et al. [9]. This sensor is designed to take in situ continuous measurements of chlorophyll fluorescence and turbidity. The sensor has a blue LED, red LED, and photodiode evenly spaced surrounding the water sample. It achieves high sensitivity by using a numeric lock-in amplifier. Preliminary turbidity measurements were made using a solution of milled flour in seawater. Although promising, the results were limited, and the precision of the turbidity measurement was not presented.

Optical fibers can also by employed in the measurement of turbidity and to detect specific organic molecules [15]. Ahmad Fairuz Bin Omar and Mohd Zubir Bin MatJafri present a good overview of the optical properties important for turbidity measurement and a design of an optical fiber turbidimeter [16]. While their design provides laboratory support for the device, the authors state that continued development is needed before it is appropriate for field measurements.

Kevin Murpty et al. also developed a low-cost autonomous optical sensor for water quality monitoring [17]. The device is similar to commercially available turbidity sondes. It contains five color LEDs and a photodiode in a cylindrical sensor body to enable spectral analysis of water quality by submerging the device. Laboratory tests and a field deployment verified the operation of the device. The measurements had high correlation with a commercial turbidity sonde. The system component cost was approximately €650 which is significantly higher than other low-cost systems proposed by others.

## 3. Appliance Sensors

**Figure 1:**
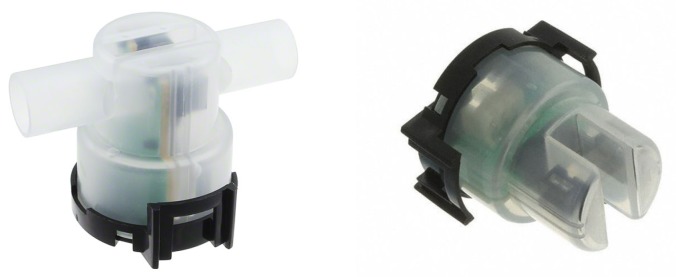
Amphenol TST-10 (**left**) and TSD-10 (**right**). TSW-10 is similar to the TSD-10 (not pictured) (images from Amphenol).

As a first step to the development of a low-cost continuous turbidity sensor, we evaluated existing commercial low-cost appliance turbidity sensors. These sensors are used in dishwasher and clothes washing machines typically to determine when the contents of the appliance are clean. It was hoped that they would be able to sufficiently determine differences in water clarity to provide useful data for water management applications. Three different turbidity sensors from Amphenol were tested (TST-10, TSD-10, and TSW-10) pictured in Figure 1. All models contain an LED emitter and a phototransistor oriented directly across (180${}^{\circ }$) from the LED. The output is proportional to the amount of light traveling through the sample and arriving at the phototransistor instead of to the measurement of the scattered light provided by a nephelometric meter. The primary difference between the various models is the mechanical enclosure. The TST-10 is a flow-through design while the TSD-10 is designed to be inserted into the water flow. Either of these could be adapted for continuous monitoring applications.

**Figure 2:**
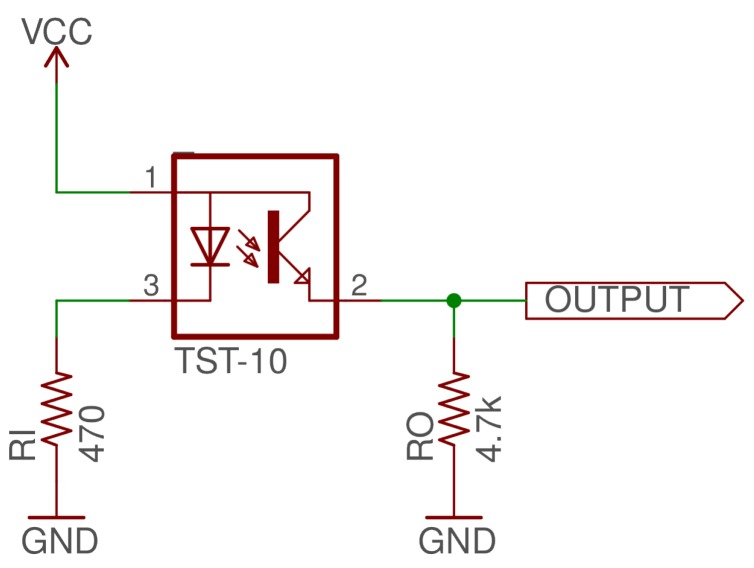
Amphenol (TST-10) appliance turbidity test circuit where VCC = 5 V. Other Amphenol models use the same circuit.

Each sensor was tested using the reference circuit specified in the datasheet [18,19,20] shown in Figure 2 and recording the voltage output of the sensor using an Arduino Mega’s internal analog to digital converter. The more light that is transmitted through the sample to the receiver the higher the output voltage. This higher voltage means the solution is clearer which is equivalent to saying that it has lower turbidity.

To test the hardware variation between sensors, we created test solutions by adding a small amount of cutting oil to water and tested four appliance sensors of the same model in the same solution. Ideally, the sensors should output the same voltage in the same solution. We performed a simple linear conversion from voltage to approximate NTU using the output curve specified in the data sheet for each sensor. Table 1 shows the observed variation between the sensors in this experiment. The result shows the actual variation is less than the worst-case value calculated from the curve in the data sheet. The TST-10 performed best with 50 NTU difference; however, for most water management applications this variation is far too large to be useful.

To improve accuracy, we can individually calibrate each sensor. According to the TST-10 datasheet, the useful range of the sensor is 0–4000 NTU with a voltage differential of 2.7 V. We used tap water (NTU ≈ 0) and recorded the sensor’s maximum voltage. The minimum voltage is specified at 4000 NTU with output voltage 2.7 V less.

To estimate the sensor’s precision, we can use a first-order linear approximation of the output over the full 4000 NTU range of the sensor. Therefore, the maximum resolution of the sensor using the Arduino’s 10-bit analog to digital converter is 7.25 NTU per analog-to-digital converter (ADC) count. As the last bit of ADC output is typically noisy, we expect the best possible result using this approach to be ±7.25 NTU with slightly better results under 1000 NTU and slightly worse results over 1000 NTU due to the non-linear output of the sensor. For most water management applications, ±1 NTU is useful, therefore, we conclude that directly connecting the sensors to the ADC cannot provide the needed resolution for water management applications even without noise or other sources of error.

## 4. Validation of the Low-Cost Nephelometric Sensor

From our previous experiments with the appliance sensors and the Arduino’s ADC, we conclude a nephelometric sensor with higher resolution ADC is necessary to achieve the precision necessary for water management applications. To explore this design space, we first constructed a sample-based sensor similar to the one developed by Kelley et al. [8].

**Figure 3:**
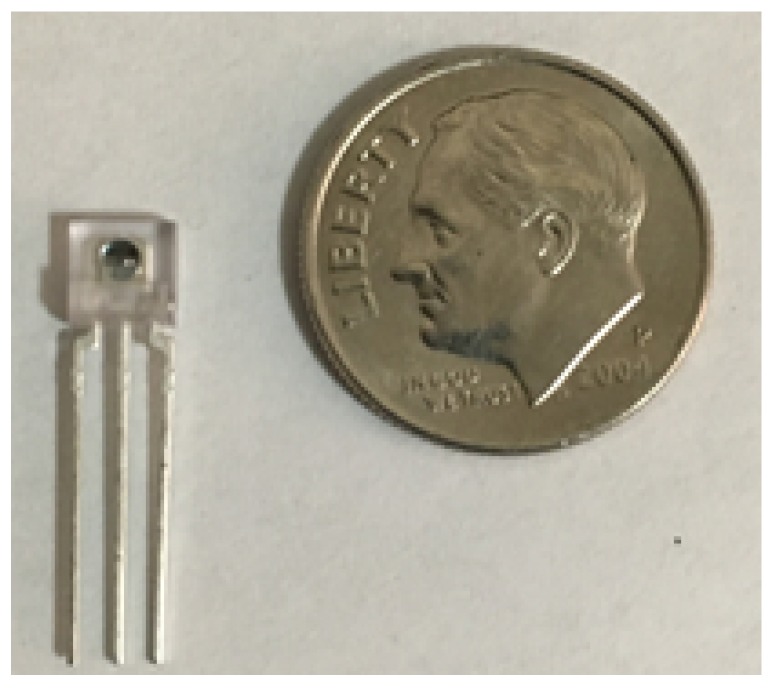
TAOS TSL235R light-to-frequency converter.

This design overcomes the ADC precision by using a TAOS TSL235R light-to-frequency converter, shown in Figure 3, to measure light intensity rather than providing an analog output. Internally the device has a photodiode sensitive to light in the range 320 nm–1050 nm. The diode current is converted to a square wave with 50% duty cycle where the output frequency is proportional to the light intensity. The range of frequencies that the converter outputs are from 0–800 kHz. Using the Arduino’s onboard Timer/Counter and Paul Stoffregen’s FreqCount library [21], we can measure the average frequency over a short interval (e.g., 1 s) with very high accuracy and precision. This approach to measuring light intensity results in far greater resolution than what is possible using the Arduino’s ADC. As a result, the sensor has a much larger dynamic range yielding higher resolution readings that are no longer strongly limited by the ADC resolution.

**Figure 4:**
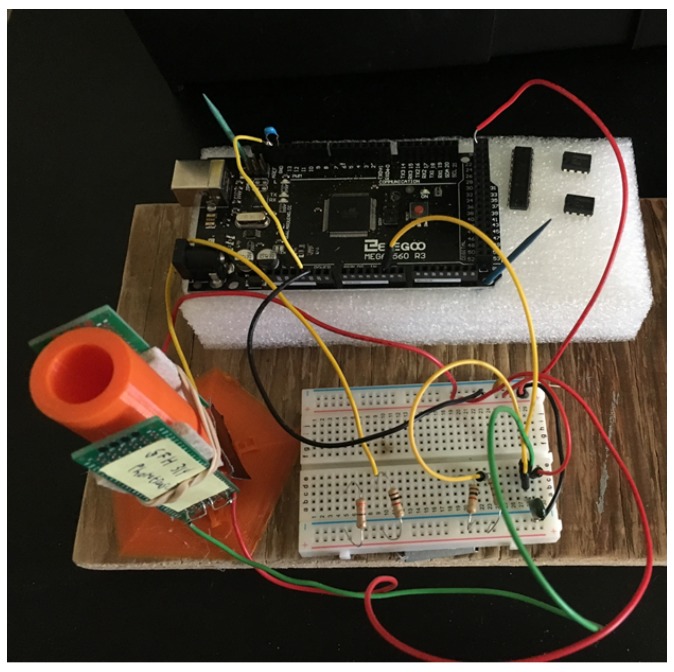
Circular sample holder and test circuit.

To evaluate the sensor, we constructed a simple test tube-based design that was 3D printed shown in Figure 4. The test tube holder allowed the 100 mA IR LED and TAOS TSL234R to be mounted securely in both 90${}^{\circ }$ and 180${}^{\circ }$ configurations. The IR LED was driven by an Arduino GPIO pin through a series 1 kΩ resistor. The frequency count was read using FreqCount on an Arduino Mega 2560.

**Figure 5:**
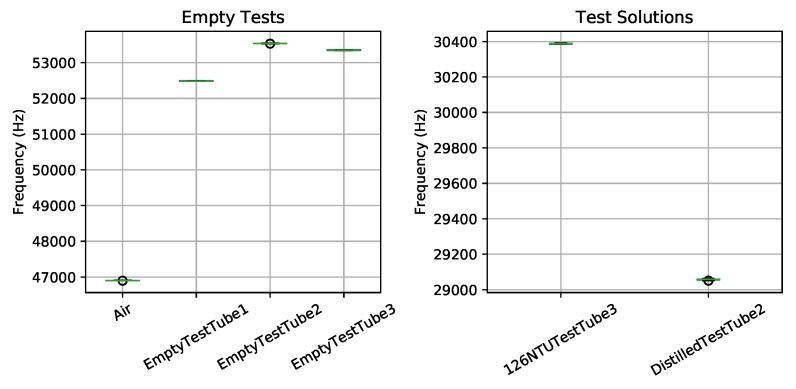
Light-to-frequency initial results using standard test tubes.

Figure 5 shows the results from several validation tests of the light-to-frequency sensor. These tests begin without a test tube inserted (Air) and three different empty test tubes. Then we test two solutions, distilled water (≈0 NTU) and a 126 NTU calibration solution. The figure shows a box plot of the measured output frequency measurements for each test on the X-axis. Each frequency measurement was generated by averaging 10 samples on the Arduino Mega and was repeated to produce 50–100 data points. From these results, we see little variation between measurements, we can clearly identify empty test tubes with frequency around 52–54 kHz, and an empty test chamber (i.e., no test tube inserted) with frequency around 47 kHz. The median of the 126 NTU calibration solution and distilled water yielded 1329.2 Hz difference. A two-point linear calibration from these values suggests sensing resolution better than 0.1 NTU per Hz may be possible (i.e., 126 NTU/1329.2 Hz = 0.095 NTU/Hz). Although further tests would need to be performed with more NTU standards to classify the accuracy and ensure a linear response.

However, these results are promising but not as good as those reported by Kelly et al. [8]. We suspect some of the error is due to the large reflections and optical impurities in the test tube. Because of the circular shape of the test tube, it is nearly impossible to keep the IR LED exactly perpendicular to its surface. As a result, we decided to switch to plastic cuvettes as they are similar but lower cost than the quartz cuvettes used by Kelly et al. [8]. Cuvettes have straight sides and are typically used in spectrophotometry where optical clarity is important.

**Figure 6:**
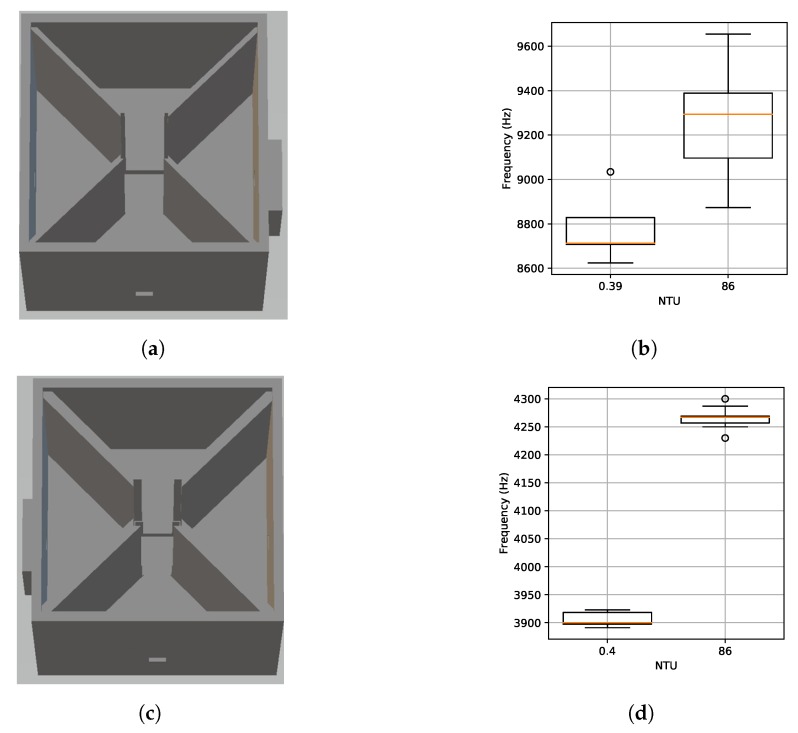
Square cuvette sample holder designs with a light-to-frequency converter at 90${}^{\circ }$ from the IR LED as well as the results from validation tests. (**a**) Initial square design. (**b**) Frequency output for initial square sample holder. (**c**) Revised square sample holder with thicker walls and tighter fit to cuvette. (**d**) Frequency output for revised square sample holder.

The housing was redesigned to have a square shape with internal walls to block any light from getting to the receiver unless it first went through the sample as shown in Figure 6a. We tested the sample holder with distilled water and a calibration solution. The test solutions were measured with a calibrated Hach 2100P turbidity meter before the experiment and measured 0.39 and 86 NTU, respectively. Figure 6b shows the observed frequency output from the light-to-frequency converter. The median difference between the samples was 581.45 Hz. Assuming a linear response, this would yield a resolution of 0.15 NTU per Hz (85.61 NTU/581.45 Hz). However, there is some overlap in the measured results between the samples and the standard deviation of the samples was large at 142.2 and 254 Hz. Moreover, this resolution is slightly worse than the test-tube-based design.

After investigation, we found that the cuvettes could rotate slightly in the sample holder and that external ambient light was causing variation in the output frequency. To rectify these problems, we revised the design to have a tighter fit to the cuvette to eliminate rotation and increased the wall thickness to reduce the effect of external light. The revised sample holder is shown in Figure 6c. We repeated the experiment with distilled water and our calibration solution. The results in Figure 6d show a significant reduction in frequency at both readings and significantly reduced variation. This result is consistent with the reduction of external light and more constant cuvette position. Although the average frequency difference was reduced to 367 Hz (0.23 NTU per Hz), the noise was greatly reduced with standard deviation of 12.0 and 19.0 Hz yielding statistically different readings in all cases.

With these results, we conclude that a sample-based low-cost nephelometric turbidity sensor using a light-to-frequency converter can provide the needed resolution needed for many water quality monitoring applications. Additionally, our revised cuvette-based sample holder successfully reduced variation in the readings. With further study and improvement, such as increasing LED brightness, we believe the performance could be improved further. This general design will be used to inform the future development of a low-cost continuous turbidity sensor.

## 5. Low-Cost Continuous Turbidity Sensing

From our previous experiments, we have validated that a low-cost nephelometric turbidity sensor can meet the requirements (i.e., better than 1 NTU resolution) needed for providing useful data for water quality monitoring applications. To provide continuous turbidity data, we will adapt the basic sensor design for flow-through applications. Many applications, such as drinking water and agriculture use commonly available pipes to transport water, such as PVC. In the U.S., schedule 40 and 80 are common specifications of PVC pipe which are available in a variety of colors and importantly for this application, clear.

**Figure 7:**
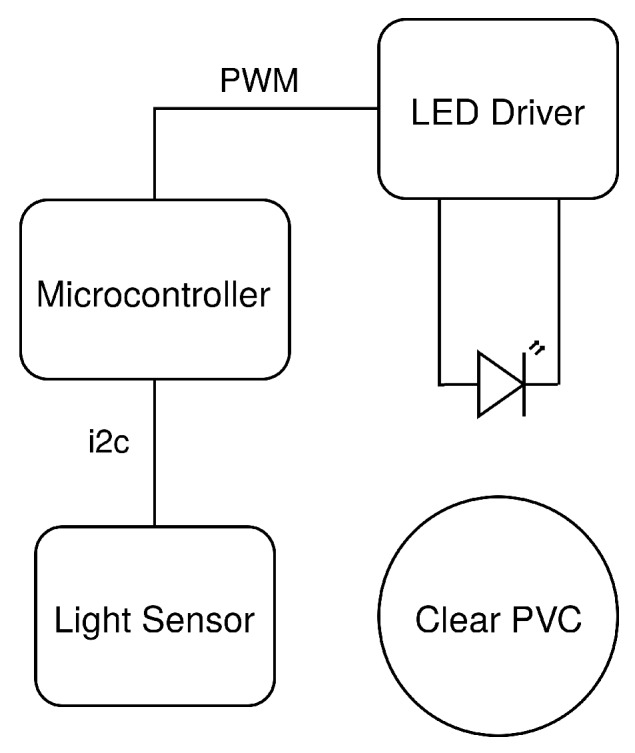
Low-cost continuous turbidity monitoring system diagram.

Our approach to the continuous low-cost turbidity monitor is to attach an LED and a light sensor on the outside of a clear PVC pipe segment oriented 90${}^{\circ }$ apart in the nephelometric configuration. In the previous tests, the separation between the LED and sensor was proportional to the width of the cuvette, which is 10 mm. In the piped configuration, this distance will be proportional to the pipe size, which could be several inches. Because the LED will be illuminating a much larger volume of water through a much thicker wall, we surmise it is useful to increase the brightness. High-powered IR LEDs (several watts) are not readily available and specialty IR LEDs are expensive. However, high-powered white LEDs are common. As a result, we replaced the IR LED with a commonly available Cree XLamp white LED (4000 K). To properly drive the LED, we use a commonly used constant current LED driver (Diodes Incorporated AL8805) configured to deliver up to 500 mA of current to the LED via a PWM control signal. This allows us to also replace the IR light-to-frequency converter with a low-cost ambient light sensor (TSL4531). These sensors are commonly used to control display brightness and provide a digital i2c output of light intensity as an integer value that is calibrated to lux. To support wireless data collection, we connect the LED driver and light sensor to an ESP32 Wi-Fi-enabled microcontroller. A diagram of the complete low-cost continuous turbidity sensing system is shown in Figure 7.

**Figure 8:**
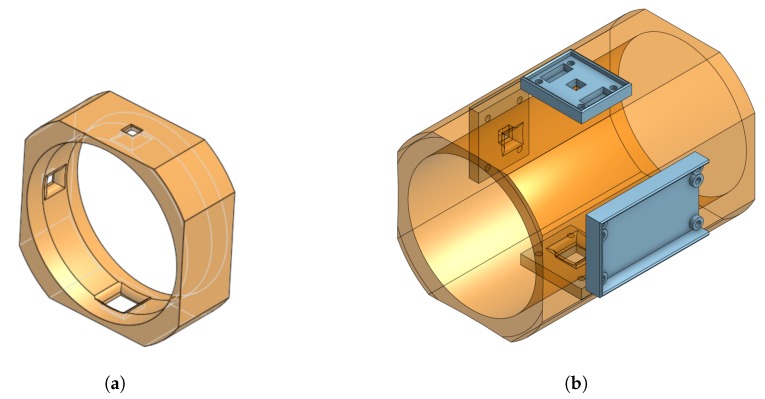
Nephelometric sensor mounts for clear pipes. (**a**) Initial design. (**b**) Revised design including PCB mounts and reduced ambient light.

To provide consistent contact with the PVC pipe, we designed a 3D-printable mounting ring to mechanically fix the LED and sensor to the pipe. Different pipe diameters can be accommodated by adjusting the dimensions of the mounting ring. Figure 8 shows a rendering of (a) our initial design and (b) revised mounting ring. With the initial design, the LED and ambient light detector were mounted to a small PCB and glued to the mounting ring. Because the PCB used through-hole connections, solder joins on the bottom of the PCB caused an uneven fit with the ring. This mechanical ring was also narrow (1 inch) and allowed ambient light to reach the light sensor. As a result, the design was revised to include PCB standoffs, recessed areas to accommodate solder joints, screw holes were added to the ring, and the height of the ring increased to block more ambient light. The mounting ring was sized to tightly fit over a section of 2-inch schedule 40 clear PVC pipe and printed in black ABS on an Ultimaker 2+ 3D printer. Black was selected to minimize reflected light in the device. Although we did not characterize this effect, we tested other colors and found black to have the lowest light level with the LED on. This suggests that reflections are minimized as desired.

The approximate unit component cost of the completed sensor is 64 USD. The component costs are shown in Table 2. This makes the low-cost continuous turbidity monitor more expensive than the affordable open-source turbidimeter ($25–$35) by Kelley et al. [8]. However, much of the cost difference is due to the WiFi microcontroller and printed circuit boards which add significant value by providing wireless communication and improved reliability over a breadboard circuit. This also compares favorably to commercial turbidity probes for water quality from manufacturers such as YSI, In situ, and Eureka where prices range from $1000 to $5000, depending on the specific model. Even compared to the low-cost autonomous optical sensor by Murphy et al. at €650 [17], we see a significant reduction in cost.

### 5.1. Laboratory Calibration

**Figure 9:**
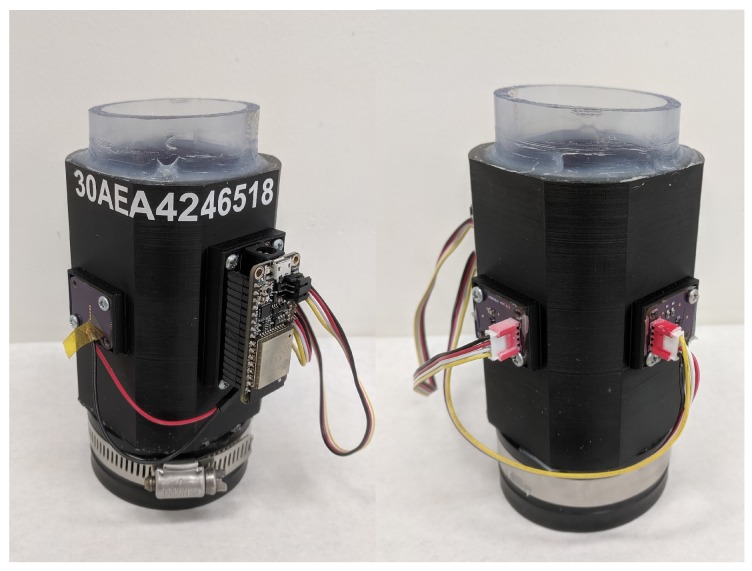
Sensor 18 configured for laboratory calibration.

Four low-cost continuous turbidity monitors were constructed and tested over the range of 0–100 NTU to explore the variation that exists in the different devices made from the same components. The devices are labeled with the last two digits of their ESP32 WiFi MAC address. For calibration, the devices were oriented vertically over a short section of clear PVC pipe with silicone caulk securing the mounting ring to the pipe and a Qwik Cap sealing the bottom as shown in Figure 9. Test solutions were added to fill the PVC pipe and a cover was placed over the top to block ambient light.

The test solutions were created by diluting a 4000 NTU formazin standard with deionized water (≈0.20 NTU) to produce solutions with values of (0.20, 5, 20, 40, and 100 NTU) [22]. The test solutions were made, and the devices were filled with deionized water and allowed to collect data for 12 h. This allowed the components and test solutions to reach a constant temperature and any air bubbles to dissipate. The devices were rinsed thoroughly with deionized water between different samples to clean any residual sample out of the pipe. Each of the samples was tested in each device for at least 10 min where the light intensity at 90-degrees, 180-degrees and the dark reading (90-degree sensor reading without the LED on) was measured every 6 s during the sampling interval. If more than 100 samples were collected for a given test solution, only the middle 100 samples were used in the analysis. Manual turbidity readings were also made of the test sample every 2 min using a Hach 2100P turbidity meter. This was done as a single reading from the meter can vary. We do not believe that the standards degraded during the testing.

After the laboratory sampling was complete, the data from each device was fit to a model of the form:

(1) $$NTU=c_{1}\times d_{0}+c_{2}\times d_{90}+c_{3}\times d_{180}+\epsilon$$

where $d_{0}$ is the light intensity at 90 degrees from the LED with the LED off in lux, $d_{90}$ is the light intensity with the LED on at 90 degrees from the LED in lux, $d_{180}$ is the light intensity with the LED on at 180 degrees from the LED in lux, and ϵ is the y-intercept. These values were computed using ordinary least squares linear regression comparing the predicted NTU to the average manual NTU reading of the sample. Models were generated for each device individually in addition to a combined model using data from all the devices. To explore the impact of each sensor (90 degrees and 180 degrees from the LED), models were generated with each sensor individually as well as both of the sensors. Table 3 shows all computed model parameters, the $R^{2}$ measure, and variance ($\sigma^{2}$) of the residual.

The value of $c_{2}$ is expected to be positive as more light reflected at 90 degrees would be more indicative of a turbid solution and all the models follow this. The value of $c_{3}$ is expected to be negative as the more turbid the solution, the less light that would pass straight through it to reach the $d_{180}$ sensor. Device 8C, 94, and B8’s models including both sensors ($d_{90}$, $d_{180}$) do not follow this expectation and have a reduced magnitude, suggesting the $d_{180}$ sensor provides inconsistent data in this NTU range.

From these results, we see that in general, the computed model fits the data well for the $d_{90}$ and $d_{180}$ as well as the $d_{90}$ only device-specific models. The variance using both sensors was slightly smaller, suggesting that the $d_{180}$ sensor does provide some information when used with the $d_{90}$ sensor. The $d_{180}$ only models have significantly larger variance when compared to the other models. The combined model also has variance that is more than an order of magnitude larger, even with both sensors, indicating there is variation between devices. This variation could be caused by the mechanical assembly construction, different brightness of the LED resulting from manufacturing differences in the LED itself or LED driver circuit, and manufacturing differences that impact the clarity of the PVC pipe. The idea of device-specific calibration is not unique to low-cost sensing. It is widely used in many manufacturing processes to compensate for errors caused by real-world manufacturing constraints without having to determine each source of variance. As a result, we omit the combined model from further analysis.

**Figure 10:**
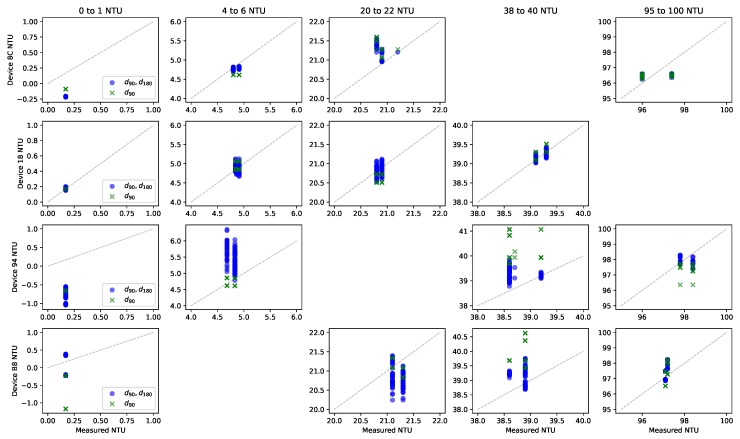
Predicted NTU vs. measured NTU for the individual models on the 5 tested NTU ranges.

To visually explore the results, Figure 10 shows several plots for the predicted NTU vs. measured NTU using the low-cost turbidity monitor. Each row presents results from a specific device while each column shows increasing NTU ranges. Missing plots result from not testing every sample on every device. The results with only the 180-degree sensor are omitted for clarity as this case performed significantly worse than the others. If the model were to produce the exact same value as the measured readings, the points would fall on the dotted diagonal line. The further the points fall from the diagonal line the larger the error of the prediction. For example, the B8 device model can predict the NTU readings for all the samples within about ±1.0 NTU. The B8 model overpredicts the NTU for the 38 to 40 NTU sample and underpredicts the NTU for the 20 to 22 NTU sample. The diagonal line bisects the plotted points for the 0 to 1 NTU sample and the 95 to 100 NTU standard, suggesting that the model, on average, predicts these cases well.

**Figure 11:**
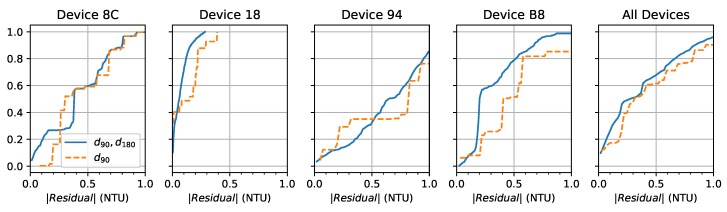
Cumulative distribution function of the absolute value of the residual by device.

To better understand these results, Figure 11 shows the cumulative distribution function (CDF) of the absolute value of the residual using the computed models. The residual is the difference between the estimated value and measured value computed individually for both sensor configurations. The range is limited to (0,1) for clarity. Residuals from all device-specific models are combined in the final “All Devices” plot. The $d_{180}$ model is not shown because it performed significantly worse than the others. We see the devices perform similarly with most of the residuals less than 1 NTU. Device 18 does the best and 94 does the worst while 8C and B8 are in the middle. For all devices, with both sensors, the median residual was −0.0032 NTU with a standard deviation of 0.4870 NTU.

**Figure 12:**
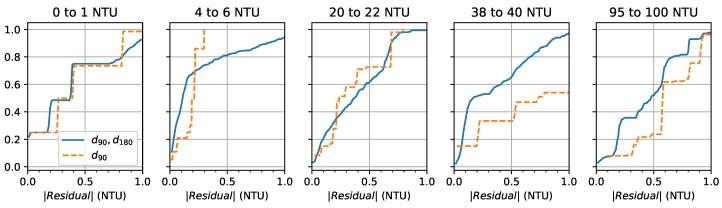
Cumulative distribution function of the absolute value of the residual by NTU range.

Figure 12 shows the CDF of the combined absolute value of the residuals from all device-specific models at each tested NTU range. This confirms that the device can be used over this whole range with consistent performance. However, this uniformity is mostly a result of performing linear regression with an equal number of data points (100) in each range. By oversampling any particular NTU range, the generated model would produce a better fit in that range at the expense of performance in the other ranges. This could be useful in certain applications to better detect small variations from an expected turbidity value.

These results show that with device-specific calibration the device will achieve accuracy closer to 1.0 NTU than our goal of 0.10 NTU over the range of 0–100 NTU. However, since the median residual was near zero, averaging multiple samples could reduce noise to approach this goal. This dataset did not have enough samples to fully investigate this question, so we will explore this in the next section.

### 5.2. Pumped Tank Test

**Figure 13:**
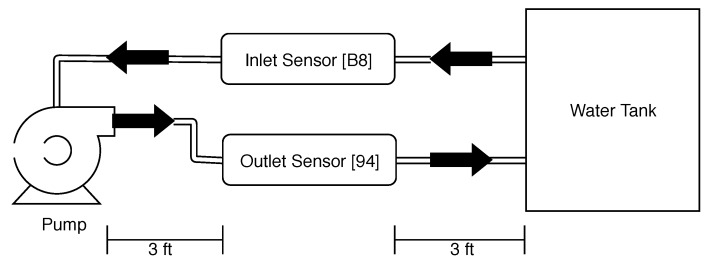
Diagram of pumped tank test.

Having calibrated and explored the performance of the low-cost continuous turbidity monitor in a laboratory setting, we now move to a simulated real-world test. For this test, we used a 1000-gallon water tank and a 1000 GPH pool pump to circulate the water. To explore if the device should be on the pump inlet or outlet, we installed a device on both. Device B8 was installed on the pump inlet and device 94 was installed on the pump outlet. Figure 13 shows a diagram of the pumped tank test. The sensors were installed using three-foot pipe segments between the tank and the pump.

The tank was filled with fresh drinking-quality water and manual turbidity measurements were made daily with the Hatch 2100P turbidimeter. These measurements were linearly interpolated between samples to produce a continuous turbidity value in the tank for analysis. The low-cost continuous turbidity monitor readings were made once every 6 s. Timestamps for each sample were recorded by the device and the clock was synchronized with a public NTP server at the start of the experiment. The timestamp and raw sensor values were then transmitted over a WiFi network to a database for storage. For analysis, the raw sensor values were linearly interpolated to a constant 1 Hz rate and a 20-min moving average of 1200 samples at 1 Hz containing about 200 raw samples was computed over 5-min periods, resulting in 288 samples per day. We chose these values to reduce the amount of data as we expect turbidity to change relatively slowly and simultaneously reduce sensor noise by averaging multiple readings. Experimentally we found that averaging over 1-min periods (10 raw samples) was sufficient to eliminate most of the sensor noise but we elected to use longer periods in our analysis to produce the desired sample rate.

**Figure 14:**
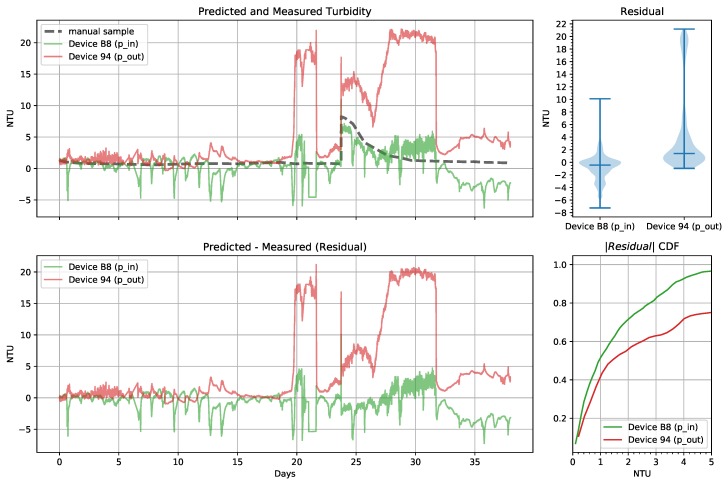
Pumped tank low-cost continuous turbidity monitor predictions from the pump inlet (p_in) and outlet (p_out) and manual measurements from a hand-held turbidimeter showing all collected data (days 0 through 37).

The filtered sensor readings were then used in the device-specific laboratory models (Table 3) to estimate the NTU reading in the tank. A small offset was present at installation, so we adjusted each device’s ϵ parameter after making the first manual reading to remove this error. Shortly after the installation, device 94 failed and the LED and light sensor was replaced with the components from device 18. Data is reported as device 94; however, the model generated by device 18 is used to predict NTU. Figure 14 shows results for 38 days of measurements. During two intervals between days 5 and 7, the data collection failed, and no samples were recorded. For analysis, the missing data were linearly interpolated between the available samples.

Initially, through day 5, the sensor on the outlet had significant noise. On day 6 we discovered that bubbles were present in the pipes near the outlet sensor and we purged the air from the pipes. On day 9 we discovered that a small hole was allowing air into the pipes. We sealed the hole and both sensors showed significantly reduced noise after this. In sealing the hole, we repositioned the outlet sensor, which caused an offset in the readings. At day 20 there was another air leak that caused a significant error and was sealed by day 22.

To investigate the response of the sensor to changing turbidity, we continued our measurements and added one quarter cup of Coffee Mate^®^ powdered coffee creamer to the 1000-gallon tank on day 23 at the pump inlet. This quickly increased the tank turbidity to about 8 NTU. The inlet sensor closely tracked this change demonstrating the impulse response of the sensor. On day 27 both sensors NTU reading begin increasing and we discovered the patch to the pipe had failed. We let this continue until day 32 when it was patched again.

The median residual and standard deviation for the inlet and outlet low-cost continuous turbidity monitors over the entire test were −0.4507, 1.9063 NTU and 1.3997, 6.5511 NTU, respectively. The cumulative distribution function of the absolute value of the residual shows that overall only slightly more than 50% of the predictions are within 1 NTU as expected from the laboratory tests even with the better performing inlet monitor. However, the large errors resulted from air in the pipes. When no air was present on days 15 through 17, the median residual and standard deviation was −0.1881, 0.2643 NTU and 0.2266, 0.1138 NTU respectively and all of the predictions were within 1 NTU with 80% of them being within 0.5 NTU.

Comparing the inlet and outlet monitors in the presence of bubbles on days 20 and 27, we see the inlet is much less sensitive to the presence of bubbles. We speculate that by going through the pump, the relatively large bubbles passing through the inlet monitor were broken up into many more small bubbles before passing through the outlet monitor. This resulted in a correspondingly larger estimated turbidity on the outlet monitor.

**Figure 15:**
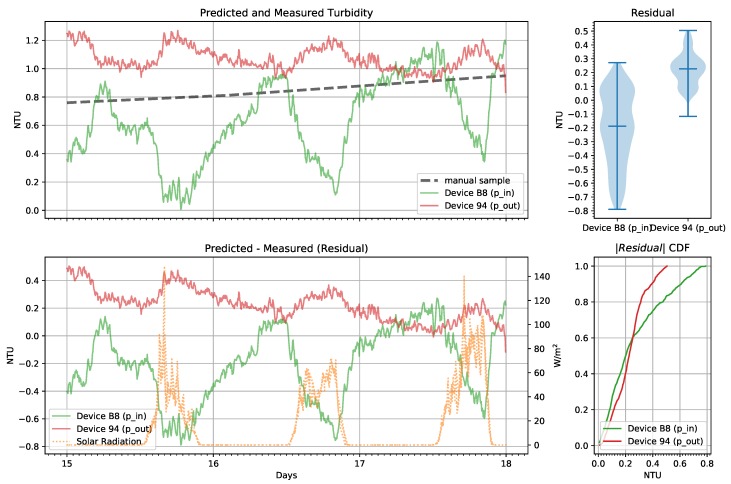
Pumped tank low-cost continuous turbidity monitor predictions from the pump inlet (p_in) and outlet (p_out) and manual measurements from a hand-held turbidimeter showing days 15 through 17.

Both sensors showed patterns of daily periodic errors. These patterns are more apparent when examining the relatively stable period between days 15 through 17, shown in Figure 15. On this plot we added the measured solar radiation (W/m${}^{2}$) from a nearby weather station for reference. The inlet sensor has a more negative residual during the day while the outlet sensor has a more positive residual and the Pearson correlation coefficients between the residual and the solar radiation was −0.58 and 0.17 respectively. To explain the difference in sign, we reexamine the model parameters from Table 3 and recall that the parameter $c_{3}$ has the opposite sign between these two devices. Furthermore, the absolute value of the parameter is larger for the inlet device. This parameter corresponds to the 180 degree sensor. As a result, we conclude that this phenomenon is almost certainly caused by ambient light. While both sensors and devices were affected, the 180 degree sensor on device B8 was the most sensitive to ambient light. The position of the devices in the test also contributed to these differences. Because our laboratory experiments were taken in relatively dark conditions, the model did not properly account for the influence of ambient light even with the dark term in the model.

### 5.3. Discussion

In this section, we describe the creation of a low-cost continuous nephelometric turbidity monitor built using commonly available components. The turbidity monitor is designed to fit over a short section of clear PVC pipe. This approach reduces the mechanical complexity of the system since no sensing components are ever in direct contact with water.

Laboratory experiments demonstrated the model was able to reliably estimate turbidity within 1 NTU with some noise present in the readings. Since the median residual was near 0, averaging multiple readings could approach our goal of 0.1 NTU resolution under well-controlled conditions.

The pumped tank test demonstrated that the sensor can continuously predict turbidity installed either on the inlet and outlet of a pump. However, the inlet sensor had better impulse response to a turbidity change. The inlet sensor showed more interference from ambient light, but we attribute this to sensor positioning and not an artifact of the pump position. The outlet of the pump was more affected by bubbles, this was attributed to the fact the pump formed some bubbles during operation. These bubbles likely dissipated when they reached the tank resulting in the inlet sensor not seeing the same level of bubbles. Overall, neither sensor achieved the same accuracy as in the lab experiment, even with averaging many sensor readings. However, on days 15–17 when no air was present, the performance of both sensors was equal to the lab experiments. By eliminating ambient light and bubbles we believe this level of performance can be maintained over longer periods. Furthermore, even at the current level of performance, many applications could benefit from low-cost continuous turbidity monitoring by detecting larger changes in turbidity (e.g., >1 NTU). Results from the last days of the experiment showed a significant offset was present suggesting that periodic calibration may be required. We plan to explore the long-term stability of the low-cost continuous turbidity monitor in future work.

## 6. Conclusions and Future Work

In this paper, we explored the development of a low-cost continuous turbidity monitor. We began by evaluating readily available appliance turbidity sensors. While inexpensive, in our tests they do not have the required accuracy for water quality monitoring applications. They were also prone to a large amount of noise and are difficult to precisely calibrate. Examining prior work on low-cost turbidity sensors, we verified that accurate low-cost sample-based turbidity sensors can be constructed. In our tests, the main source of error was the imprecision of the sample holder (Cuvette or Test Tube) in the sensor apparatus. Using this design as a starting point, we adapted the sensor for use in piped-water applications. Lab tests verified that with individual calibration, accuracy better than 1 NTU is possible over the range 0–100 NTU. A 38-day long experiment was performed with the low-cost continuous turbidity monitor in a piped-water application. The monitor showed more error than in the lab experiments, yielding ≈5 NTU accuracy and good response to changes in turbidity. The primary source of error was attributed to bubbles in the liquid and ambient light. This may be sufficient for some continuous monitoring applications. For other applications where higher accuracy is needed, we believe that by reducing ambient light on the sensor and eliminating all air from in the pipes will yield accuracy better than 1 NTU. Like all other turbidity sensors, periodic calibration is necessary to maintain the accuracy of the low-cost continuous turbidity monitor.

As we found that device-specific calibration significantly improves performance, a simpler way to calibrate the sensor is recommended as lab-made turbidity standards are not commonly available by citizen scientists. There are other processed liquids that have consistent turbidity such as apple juice and tea which could be used for calibration. A validated procedure to calibrate the sensor with these liquids could be developed. We also plan longer trials to verify the long-term behavior of the low-cost continuous turbidity monitor. One long-term concern is if and when to remove and clean the clear PVC section. Since PVC can develop a static charge, contaminants may be attracted to the clear pipe segment. It is not clear if the pumped liquid is sufficient to remove these contaminants. We plan to redesign the mounting ring to simplify removal for inspection and cleaning.

## Figures and Tables

**Table 1:** Variation between the appliance sensors of the same model.

Sensor	Specified Variation (NTU)	Observed Variation (NTU)
TSD-10	305	162
TST-10	325	50
TSW-10	748	348

**Table 2:** Unit component cost of the prototype sensor.

Component	Approximate Cost (USD)
ESP32 microcontroller	20
TSL4531 light sensor	1
XLamp MX-6 LED (2)	2
Printed circuit boards	10
PVC pipe 2 in × 12 in	16
Black ABS filament (83 g)	5
Miscellaneous components	10
Total	64

**Table 3:** Individual and combined model parameters, $R^{2}$, and residual variance ($\sigma^{2}$) for using the 90- and 180-degree sensors, only the 90 degree, and only the 180 degree sensor respectively. The units of $c_{1}$, $c_{2}$ and $c_{3}$ are NTU/lux. The unit of ϵ and $\sigma^{2}$ are NTU and NTU${}^{2}$ respectively.

Device	Sensor (s)	$c_{1}$	$c_{2}$	$c_{3}$	ϵ	$R^{2}$	$\sigma^{2}$
Device 18	$d_{90}$, $d_{180}$	−0.2956	0.1608	−0.0046	41.2997	1.0000	0.0093
Device 18	$d_{90}$	−0.2970	0.2088	0.0000	−16.0266	0.9999	0.0316
Device 18	$d_{180}$	−0.3372	0.0000	−0.0200	232.7049	0.9989	0.2582
Device 8C	$d_{90}$, $d_{180}$	0.1112	0.2313	0.0018	−38.2779	0.9998	0.2446
Device 8C	$d_{90}$	0.1055	0.2136	0.0000	−15.4716	0.9998	0.2570
Device 8C	$d_{180}$	0.3477	0.0000	−0.0212	259.3874	0.9984	2.3556
Device 94	$d_{90}$, $d_{180}$	−0.0995	0.3770	0.0117	−174.0959	0.9996	0.5432
Device 94	$d_{90}$	−1.1277	0.2398	0.0000	−17.6853	0.9993	1.0413
Device 94	$d_{180}$	−2.5629	0.0000	−0.0204	254.4228	0.9972	4.2926
Device B8	$d_{90}$, $d_{180}$	−0.5757	0.3274	0.0059	−100.8465	0.9999	0.1515
Device B8	$d_{90}$	−0.9409	0.2537	0.0000	−21.2899	0.9997	0.4269
Device B8	$d_{180}$	−1.9002	0.0000	−0.0201	251.4739	0.9957	5.5707
Combined	$d_{90}$, $d_{180}$	1.6055	0.2252	−0.0003	−13.6549	0.9934	8.0515
Combined	$d_{90}$	1.6541	0.2286	0.0000	−17.8185	0.9934	8.0627
Combined	$d_{180}$	1.2328	0.0000	−0.0202	246.3589	0.9558	53.9348
